# Turning Cancer Immunotherapy to the Emerging Immune Checkpoint TIGIT: Will This Break Through the Limitations of the Legacy Approach?

**DOI:** 10.3390/vaccines12121306

**Published:** 2024-11-22

**Authors:** Haozhe Cui, Eyad Elkord

**Affiliations:** 1 Department of Biosciences and Bioinformatics, School of Science, Suzhou Municipal Key Lab in Biomedical Sciences and Translational Immunology, Xi’an Jiaotong-Liverpool University, Suzhou 215123, China; haozhe.cui24@student.xjtlu.edu.cn; 2 College of Health Sciences, Abu Dhabi University, Abu Dhabi 59911, United Arab Emirates; 3 Biomedical Research Center, School of Science, Engineering and Environment, University of Salford, Manchester M5 4WT, UK

**Keywords:** immune checkpoint, TIGIT, PD-1, antibody development, small-molecule therapy

## Abstract

The discovery of immune checkpoints (ICs) has pushed cancer treatment into the next era. As an emerging immune checkpoint, the TIGIT/CD155 axis inhibits the cytotoxicity of T and NK cells through multiple pathways. Immune checkpoint inhibitors (ICIs) targeting TIGIT are hopefully expected to address the issue of unresponsiveness to anti-PD-(L)1 monoclonal antibodies (mAbs) by combination therapy. This paper presents insights on the expression, structure and mechanism of action of TIGIT, as well as the principles and methods of designing mAbs targeting TIGIT and their clinical data. The advantages and disadvantages of targeting TIGIT using mAbs, bispecific and tri-specific antibodies (bsAbs and tsAbs), peptides, and compounds, in addition to potential combination therapies of anti-TIGIT with anti-PD-1 or cancer vaccines, are addressed. Finally, perspectives on current immunotherapies targeting TIGIT are discussed.

## 1. Introduction

According to a report in The Lancet, the factors affecting human mortality have essentially been the same from 1990 to the present. Excluding the impact of COVID-19 on human deaths in 2019–2021, the data show that cancer is one of the leading causes of mortality, along with cardiovascular disease and chronic obstructive pulmonary disease (COPD) [1]. The traditional approach against tumors is to remove the diseased tissue through surgery, as well as radiation and chemotherapy, but the limitations are also clear; they are only effective in the early stage of the disease and have potential to cause multiple side effects by damaging autologous healthy cells [2,3]. With the development of molecular biology, a significant milestone in tumor therapy is shifting the focus from tissues and organs to molecular mechanisms and the tumor microenvironment (TME) [4]. The discovery of immune checkpoints (ICs) and the great success of their inhibitors have pushed cancer research even more towards immunotherapy. Homeostasis is extremely important in any biological system, and the immune system is no exception. An over-activated immune system can lead to autoimmune diseases; therefore, mammals have evolved some immunosuppressive mechanisms, and ICs are an impressive class in this regard [5,6]. The binding of this class of receptors and ligands inhibits immune cell activity through a variety of mechanisms. The first two ICs to be discovered were programmed cell death 1 (PD-1) and cytotoxic T lymphocyte-associated antigen 4 (CTLA-4), and their positive clinical data and great potential in immunotherapy led to the award of the Nobel Prize in Physiology or Medicine to James P. Allison and Tasuku Honjo [7]. Immune checkpoint inhibitors (ICIs) can block the binding of IC molecules to their ligands, thereby restoring T/NK cell cytotoxicity or activity. The most extensively investigated and developed ICIs are monoclonal antibodies (mAbs), and currently they are the only type of approved drugs on the market [8]. FDA-approved antibodies include ipilimumab and tremelimumab (targeting CTLA-4), nivolumab, pembrolizumab, cemiplimab, atezolizumab, avelumab and durvalumab (all targeting the PD-1/PD-L1 axis) [8,9]. PD-1/PD-L1 antibody drugs are superior in both range of use and less side effects compared to CTLA-4 antibody drugs. Positive effects of these mAb drugs have been observed in the clinical management of a wide range of tumors, and most encouragingly, some patients have demonstrated complete remission [10]. However, the overall treatment response rate is unsatisfying, with approximately four-fifths of patients failing to respond, and it may be influenced by resistance mechanisms such as insufficient tumor antigenicity [11]. Thus, enthusiasm for the discovery and study of novel immune checkpoints has been undiminished.

**Figure 1:**
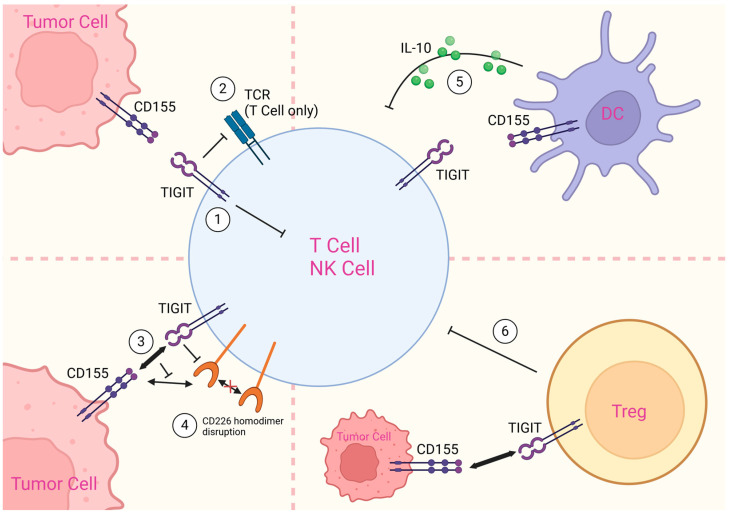
**Mechanisms of immune cell suppression by TIGIT.** ① TIGIT delivers inhibitory signals to NK cells via its cytoplasmic tail. ② TIGIT inhibits TCR-mediated T cell activation. ③ TIGIT competes with activating receptor CD226 for their co-ligand CD155. ④ TIGIT disrupts homodimerization of CD226. ⑤ CD155 on DC binds to TIGIT and induces secretion of IL-10 to inhibit T/NK cell responses. ⑥ Tregs upregulate TIGIT, which binds to CD155 on tumor cells, generating a more suppressive phenotype that can inhibit T/NK cells. Created in https://BioRender.com.

T-cell immunoreceptor with immunoglobulin and tyrosine-based inhibitory motif domain (TIGIT), also called WUCAM, Vstm3 and VSIG9, was first identified in 2009 through genome-wide search strategies [12]. Unlike PD-1, TIGIT expression is strictly limited to CD8^+^/CD4^+^ T cells and T regulatory cells as well as innate lymphoid cells (ILCs) including natural killer (NK) cells and γδ T cells. TIGIT expression is upregulated during T cell activation, so it can hardly be detected on non-activated CD8^+^/CD4^+^ T cells, but can be induced quickly after T cell activation. Effector T cells and memory T cells express higher levels of TIGIT than naïve T cells [12,13]. TIGIT belongs to the PVR/nectin family, and it contains an immunoglobulin variable domain, a transmembrane domain, and an intracellular signaling region. The intracellular portion contains an immunoreceptor tyrosine-based inhibitory motif (ITIM) and an immunoglobulin tyrosine-tail (ITT)-like motif, which is crucial for inhibitory signaling [12,14,15,16]. TIGIT has four cell membrane-localized ligands: CD155, CD112, CD113 and Nectin-4 [17,18,19], and one ligand secreted by bacteria: Fap2 protein [20]. Among those, CD155 has the highest affinity, and it was previously identified as the poliovirus receptor (PVR) [21,22]. A recent study using single-cell RNA sequencing confirmed that the TIGIT-CD112 axis is a key immune checkpoint in neuroblastoma, but more studies on blocking ligands other than CD155 are still needed [23]. The mechanisms by which TIGIT suppresses the immune system are shown in Figure 1.

CD226 is also known as DNAX accessory molecule-1 (DNAM-1), and is a co-stimulatory receptor that activates cytotoxicity in immune cells. CD226 is expressed more broadly than TIGIT. In addition to the previously described cells including CD8^+^/CD4^+^ T cells, NK cells and Tregs, CD226 has been observed in a number of additional immune cell types, including NKT cells, B cells, macrophages and dendritic cells. It has also been identified in hematopoietic precursor cells, megakaryocyte/platelet lineage, endothelial cells and mast cells [24]. The binding of CD155 or CD112 activates CD226, which transmits activation signals through its cytoplasmic tail to stimulate T/NK cells via a signaling pathway in the following order: CD226 activation–lymphocyte function-associated antigen 1 (LFA-1)–intracellular adhesion molecule 1 (ICAM-1)–Fyn-Akt signaling pathway [25,26]. Furthermore, the binding of CD226 to CD155 results in the phosphorylation of FOXO1, which is then translocated from the nucleus to the cytoplasm and ubiquitinated. This process enhances the activity of NK cells and effector/helper T cells [27,28]. Through competitive binding to the CD155 and CD112, TIGIT blocks the stimulatory signaling of the immune-activated receptor CD226 and has the ability to disrupt its homodimerization, which is known as a major immunosuppressive mechanism of TIGIT [29,30]. In addition, TIGIT sends inhibitory signals directly to NK cells through its cytoplasmic tail and inhibits TCR-driven activation signals to directly suppress T-cell cytotoxicity [31,32]. Dendritic cells (DCs) and T regulatory cells (Tregs) are also involved in the process of immune cell suppression by TIGIT. CD155 expressed by DCs accelerates the secretion of IL-10 to indirectly inhibit T cell cytotoxicity upon binding to TIGIT [12], whereas an increase in the expression of TIGIT on Tregs is more favorable for their conversion to an immunosuppressive phenotype [33]. The discovery of these immunosuppressive mechanisms has prompted thinking about the possibility of targeting TIGIT in cancer therapy, and while the main approach has been the development of monoclonal antibodies, there are some less-investigated approaches, such as the use of small-molecule inhibitors.

## 2. Co-Inhibition Mechanisms of TIGIT and PD-(L)1

Although PD-1 blockade is currently the most widely clinically used IC blockade therapy, response rates are insufficient. There are a number of factors that contribute to anti-PD-1 resistance, which occurs predominantly in immunosuppressive TME, including absence of tumor antigens, insufficient antigen presentation, immunosuppressive molecules, and insufficient T-cell infiltration [34]. Despite sufficient antigenicity, the efficacy of PD-(L)1 inhibition may be undermined by the inadequate response of tumor cells to IFN-γ signaling and the tumor-intrinsic loss of MHC [11]. On T cells, TIGIT is strongly correlated with PD-1 expression, and it has been observed that inhibition of PD-1 significantly increases TIGIT expression on the surface of cytotoxic T cells, which is regarded as a compensatory mechanism between ICs [30,35,36]. This inspired mechanistic investigations into the synergistic effects of PD-1 and TIGIT. As mentioned previously, TIGIT competes with CD226 for ligands, thereby inhibiting CD226. PD-1 synergistically inhibits CD226 at the intracellular level. Specifically, CD226 signaling is dependent on phosphorylation of its cytoplasmic tail, whereas PD-1 binds to the ligand and its intracellular structural domain recruits Shp2 to inhibit CD226 phosphorylation [36]. This suggests that CD226 is an important intermediate linking TIGIT and PD-1 blocking therapies. Clinical trials confirmed effectiveness of TIGIT/PD-(L)1 co-blockade; co-blockade therapies showed better results compared to nearly zero objective remission rates (ORRs) with anti-TIGIT monotherapy and were also better than using anti-PD-1 mAbs alone [37,38]. A recent study combined bulk RNA-seq and multiplex immunofluorescence (mIF) of pre-treatment tumor samples from the clinical trial CITYSCAPE, as well as analysis of on-treatment serum samples and peripheral blood mononuclear cells (PBMCs). The results showed that improved efficacy of combination therapy is strongly associated with high levels of macrophages, monocytes and Tregs in pre-treatment samples. Their preclinical model suggests that anti-TIGIT (tiragolumab) may activate myeloid cells and synergize with anti-PD-L1 antibodies through the binding of its functional Fc segment and the Fcγ receptor (FcγR) on myeloid cells [39]. This is the first demonstration of the beneficial role of the Fc segment of the TIGIT antibody and myeloid cell participation in TIGIT/PD-(L)1 combination therapy.

## 3. Monospecific mAbs Against TIGIT and Co-Inhibition Therapy

TIGIT possesses multiple mechanisms in immunosuppression involving direct inhibitory signaling and antigen-presenting cell (APC)/Treg-mediated suppression, which shows huge potential for drug development [17,18,24]. In order to target this promising checkpoint in cancer immunotherapy, the most straightforward approach would be the use of antibodies, because they are relatively easy to develop. The protein–protein interactions of TIGIT with natural ligands make it possible to develop antibodies that occupy the binding site or spatially prevent ligand binding after antigen–antibody combination. Katharina and her co-workers have resolved the structure of TIGIT and its primary ligand CD155 binding form [40]. TIGIT and CD155 bind and form a homodimer with no significant conformational changes during the binding process. At the molecular binding site, a typical lock-and-key structure locks the two molecules together, with the lock consisting of the conserved AX_6_G motif and the keys being Y113 in TIGIT and F128 in CD155 [40]. The crystal structure of the tetramer suggests that TIGIT-CD155 signaling appears to be dependent on signal cluster formation mediated by a cis-anti-aggregation mechanism [40]. The designing principle of antibodies against TIGIT requires receptor–ligand blockade; thus, most existing mAbs are developed based on IgG1 because their Fc segment is active in antibody-dependent cellular cytotoxicity (ADCC) and complement-dependent cytotoxicity (CDC) [41,42]. When developing anti-TIGIT antibodies, it is essential to consider several factors, including complementarity-determining region (CDR) screening, humanization and affinity maturation. Phage display technology is commonly used for core sequence screening in anti-TIGIT mAbs development. This technique screens for antibodies with high antigenic affinity by constructing human immune libraries, fusing these antigen-binding fragments (Fabs) or single-chain variable fragments (ScFvs) to pIII surface proteins and thus displaying them on the phage surface, followed by reverse transcription to obtain the gene sequence [43].

Table 1 lists current promising anti-TIGIT mAbs; they were selected because they went further in clinical trials or due to their available binding structures. A large number of clinical trials using a combination of anti-TIGIT antibodies and anti-PD-(L)1 antibodies are ongoing [17]. This article presents the two most important clinical trials with anti-TIGIT mAbs involved. The efficacy of using vibostolimab alone and in combination with pembrolizumab is evaluated. Patients were divided into two groups, group A with advanced solid tumors (dose escalation and dose confirmation phase) and group B (expansion phase in specific tumor types) with non-small cell lung cancer (NSCLC); both groups received either monotherapy or combination therapy. In group A, the proportions of treatment-related adverse events (TRAEs) were higher in patients with the combination therapy (62%, with the most common being fatigue and itching) than patients treated with a single agent (56%, with the most common being pruritus and rash); grade 3–4 TRAEs are 17% and 9%, respectively [37]. Combination therapy has a clear advantage in the confirmed overall remission rate (ORR) at 7%, while the ORR is 0% in single-agent therapy. In group B, patients without prior anti-PD-(L)1 therapies received combination therapy (confirmed ORR 26%) and PD-(L)1-refractory patients received monotherapy and combination therapy (both confirmed ORR 3%). In combination therapy, the most common TAREs are pruritus, hypoalbuminaemia and fatigue, while they are rash and fatigue in monotherapy [37]. Another mAb drug, tiragolumab, has a full Fc segment and its efficacy was evaluated in a clinical trial in combination with atezolizumab. The group treated with tiragolumab and atezolizumab combination therapy (31.3%) showed a significantly higher objective response rate compared to using atezolizumab alone (16.2%). In terms of serious TRAEs, the combination therapy group was 3% higher than atezolizumab alone [38]. However, the phase III clinical trial of tiragolumab in combination with atezolizumab did not meet its co-primary endpoint of PFS; tiragolumab did not enhance the efficacy of anti-PD-1 mAb in combination with carboplatin and etoposide (CE) in untreated extensive-stage small cell lung cancer (ES-SCLC) [44]. It is not surprising to find that the ongoing clinical trials of anti-TIGIT mAbs are all in combination with anti-PD-(L)1 antibodies; this is because the clinical application of anti-TIGIT mAbs as a monotherapy did not yield anticipated results, despite the promising outcomes that have been observed in the laboratory. In addition, co-suppression of the two immune checkpoints, TIGIT and PD-(L)1, has a molecular basis. TIGIT competes for ligands with the activating receptor CD226 extracellularly and disrupts its homodimerization, while PD-1 inhibits CD226 phosphorylation via its ITIM motif intracellularly after binding PD-L1 [45]. Overall, using anti-TIGIT mAbs in combination with anti-PD-1 mAbs provided higher efficacy as well as manageable side effects, especially for anti-PD-1 resistant patients. Moreover, blocking TIGIT not only restores T-cell activity, but also increases immune killing through immune-promoting DCs and attenuated Treg immunosuppression, which has great potential compared to those single-pathway immune checkpoints. However, mechanisms of resistance to immune checkpoint mAbs and compensations between co-inhibitory molecules cannot be ignored. TIGIT blockade therapies rely on activated T and NK cells; however, the interaction of cancer cells, stromal cells and immune cells in the complex TME promotes chronic inflammation and immunosuppression [46]. Inhibition of several immunosuppressive molecules may contribute to the upregulation of other immunosuppressive molecules, which needs to be addressed. In addition to immune checkpoints like PD-(L)1, co-inhibition of TIGIT with other molecules such as IDO may be considered [47]. Unlike PD-1 inhibition, a validated biomarker for TIGIT inhibition has not yet been identified, which makes it more difficult to judge the response rate of co-inhibition clinically [48].

## 4. Alternative Methods for TIGIT Inhibition

As a result of the elucidation of the mechanism of co-inhibition, researchers have developed bispecific antibodies (bsAbs). According to a recently published study, NSWh7216 is a bsAb that targets both human CD25 and TIGIT, preferentially targeting Tregs expressing high levels of both CD25 and TIGIT, as an increased number of CD25/TIGIT double-positive Tregs in the solid tumor microenvironment were observed [50]. NSWh7216 treatment selectively depletes intratumoral Tregs rather than peripheral Tregs, as peripheral Tregs express lower levels of CD25 and TIGIT. It demonstrated a notable capacity for tumor killing in CD25 humanized mice [50]. Oricell Therapeutics developed BiPT-23 bsAb by cloning anti-TIGIT variable domain of heavy-chain antibody (VHH) onto the N-terminal of the heavy chain of YN035 (anti-PD-L1 mAb) and demonstrated its stability and cytotoxicity. BiPT-23 significantly inhibited tumor growth in vivo while depleting Tregs without affecting CD11b^+^F4/80^+^ DCs and macrophages within the TME [51]. In addition to PD-(L)1, TIGIT also serves as a co-target along with lymphocyte activation gene-3 (LAG-3) in antibody designing. ZGGS15 is a bsAb against TIGIT and LAG-3, which competitively inhibits their binding to ligands (CD155 and major histocompatibility complex class II, MHC-II). It was designed as an IgG4 isotype to circumvent ADCC or CDC as well as to enhance interaction with FcRn. Increased anti-tumor efficacy of ZGGS15 over monospecific anti-TIGIT or anti-LAG-3 mAbs has been demonstrated in vivo and in vitro [52]. ZGGS15 demonstrated the potential of TIGIT and LAG-3 co-inhibition; however, the precise molecular mechanisms involved remain to be elucidated. In addition, its anti-tumor effect in vivo was not compared with the combination of anti-TIGIT and anti-LAG-3 antibodies; thus, more studies are needed to demonstrate the necessity of using a bsAb rather than two monospecific mAbs. GB265 (PDL1 × TIGIT bsAb), GB266 (PDL1 × LAG3 bsAb) and GB266T (PD-L1 × TIGIT × LAG-3 tri-specific antibody, tsAb) were developed together by phage display and computer-aided antibody design (CAAD). They all have superior T-cell activation and tumor killing capabilities than the use of benchmark antibody combinations, both at the cellular level and in vivo [53]. Unlike ZGGS15, their Fc segment is fully functional and promotes anti-tumor effects [53]. The function of the Fc segment of anti-TIGIT mAb has been supported by further research, possibly with similar mechanisms [39]. Bispecific or tri-specific antibodies seem to have potential, as they not only inhibit their targets simultaneously, but also set bridges between immune cells and tumor cells. However, they do not address specific limitations of antibody-based drugs, such as poor tissue penetration. Small-molecule drugs provide a new avenue. The two teams that developed the widely known d-peptide ^D^PPA-1, which targets PD-1, have teamed up again to develop the ^D^TBP-3 targeting TIGIT [54,55]. In order to screen for D-peptides targeting TIGIT using mirror phage display technology, the researchers first synthesized the D-amino acid version of TIGIT IgV in four segments. This was subsequently used as a source to screen for affinity L-peptides and then converted to D-peptides. Molecular docking showed that ^D^TBP-3 occupies the binding pocket of CD155; in the CT26 mouse tumor model, ^D^TBP-3 inhibited tumor growth in a T cell-dependent manner [54]. Since ^D^TBP-3 is a short peptide, it solves the disadvantage of poor penetration of mAb proteins; meanwhile, it has lower immunogenicity than mAbs, which may facilitate their efficacy. Using D-peptide is a double-edged sword; despite prolonging the half-life of the drug and increasing its stability in human serum, it may also increase the toxicity of the drug. The metabolic and organ toxicity of this drug needs to be considered carefully, although the authors stated that ^D^TBP-3 accumulates in the liver and kidneys and is therefore not a cause for concern [54]. To our best knowledge, only one full D-peptide has been approved by the FDA [56]. In addition to peptides, several chemical compounds that act on TIGIT have been developed, including elraglusib [57], Gln (TrT) [58] and hemin [59]. Elraglusib is known as an inhibitor of glycogen synthase kinase-3; it has been shown to reduce the expression of immune checkpoint molecules TIGIT, PD-1 and LAG-3, and to enhance melanoma cell killing by CD8^+^ T cells either as a single agent or in combination with anti-PD-1 mAb [57]. Gln (TrT) is an interesting small-molecule compound that can block both TIGIT/PVR and PD-1/PD-L1 interactions, and the antitumor effects of this molecule have been demonstrated at the cellular level and in the MC38 model [58]. Hemin was screened by virtual molecular docking, and it was shown that it can block TIGIT/CD155 interaction and interact with elevated IFN-γ to induce iron death in tumor cells [59]. These chemical compounds showed good anti-tumor properties and are cheap and easy to mass produce and transport, but the uncertainty associated with off-target effects needs to be considered. These alternative methods targeting TIGIT will prove flexible enough to be used in combination with other methods, such as combined PD-(L)1 blockade, CAR-T or cancer vaccines.

## 5. TIGIT, PD-1 and Anti-Cancer Vaccine

Cancer vaccine therapy is based on the delivery of tumor-derived antigens to the immune system to induce an immune response against specific tumor-associated antigens (TAAs). The identification of tumor-specific antigens can enhance the safety of cancer vaccines by enabling activated immune cells to target cancer cells specifically, avoiding the inadvertent destruction of normal cells. However, cancer vaccines have challenges in efficacy, because tumor heterogeneity and immunosuppressive TME result in poor response [60,61]. Thus, combining cancer vaccines and ICIs may lead to a breakthrough. Immune checkpoint inhibitors are T/NK cell-dependent and are usually effective in cancers with high immune cell infiltration. In contrast, cancer vaccines increase the number of T cells targeting the tumor by delivering tumor antigens. In turn, ICIs resist T-cell depletion in the TME. Two studies evaluated the impact of PD-1 and TIGIT co-inhibition on cancer vaccine therapy in tumor-bearing mice; the results showed that the combined blockade of TIGIT and PD-1 reversed T-cell depletion due to upregulated PD-L1 and CD155, showing better anti-tumor effects than using cancer vaccine alone [62,63]. Notably, the use of cancer vaccines in ‘cold’ TC1 tumors greatly enhanced the efficacy of TIGIT/PD-1 co-inhibition [62]. The above data suggest that the synergistic mechanism of TIGIT/PD-1 co-inhibition and cancer vaccines is promising to break through their respective limitations and bring new hope for immunotherapy.

## 6. Perspective

The discovery of immune checkpoints has significantly advanced the field of immunotherapy. Compared to conventional therapies, immune checkpoint blockade is a safe and highly effective treatment for non-solid tumors [64]. As an emerging immune checkpoint, TIGIT has great potential in the treatment of tumors resistant to PD-(L)1 inhibitors, with the molecular mechanism of co-inhibition elucidated and confirmed in clinical trials [36]. Blockade of TIGIT and other immune checkpoints relies on the activated immune cells to eradicate tumors, which means that tumor-infiltrating lymphocytes (TILs) play a pivotal role in this process, thereby limiting the applicability of this therapy in immunosuppressed patients and ‘cold’ TME [8]. A combination of anti-TIGIT/PD-1 therapy with treatment to convert ‘cold’ TME into ‘hot’ TME could be considered. For example, the direct delivery of drugs into tumor tissue and the subsequent induction of local inflammation in the diseased tissue can enhance the infiltration of immune cells, thereby facilitating the efficacy of immune checkpoint blockade therapy [65]. In addition, there is a lack of validated biomarkers that can predict anti-TIGIT therapy. Some labelled peptides have recently been reported as promising biomarkers in PET imaging [66,67], and it is expected that more validated biomarkers predictive of anti-TIGIT therapy will be identified. When designing small-molecule inhibitors targeting TIGIT, in addition to blocking the interaction between TIGIT and the ligand, attempts can also be made to inhibit TIGIT transcription and expression [10]. When considering immunotherapy, TIGIT/CD155/CD226 should be viewed as a dynamic immunoregulatory axis; their interactions connect a larger immunoregulatory network with other ICs and receptors. Antibodies targeting CD155 have not been shown to be effective in clinical trials [68], possibly due to (1) concomitant blockade of extracellular signaling from CD226, (2) multiple immunosuppressive pathways of TIGIT, which means that only blocking CD155 does not prevent TIGIT from disrupting homodimerization of CD226, and binding with other ligands continues to exercise TIGIT cytoplasmic tail function, (3) overexpression of CD155 in a wide range of tumors and the potential to express soluble CD155 (sCD155), causing difficulty in controlling the dosage and frequency of drug administration. Oncolytic polio virotherapy seems to have potential, because it induces lysis of tumor cells to release danger-associated molecular patterns (DAMPs) that activate NK and T cells, and its targets are highly expressed in tumors [69], but efficacy needs to be demonstrated clinically.

## Figures and Tables

**Table 1:** Promising monoclonal antibodies targeting TIGIT.

Antibody Name	Fc Type	Clinical Trial	Developer	Binding Structure
Vibostolimab	Active	Phase III	Merck	Unanalyzed
Tiragolumab	Active	Phase III	Genentech	Analyzed (PDB: 8JEO)
Domvanalimab	Inactive	Phase III	Gilead	Unanalyzed
Etigilimab	Active	Phase II	Mereo BioPharma	Unanalyzed
Ociperlimab	Active	Phase III	BeiGene	Analyzed (PDB:8JEL, 8JEN)
MG1131	Inactive	None	Jeong et al. [49]	Analyzed (PDB: 7VYT)

PDB: RCSB Protein Data Bank (https://www.rcsb.org/).
